# Reducing Phthalate, Paraben, and Phenol Exposure from Personal Care Products in Adolescent Girls: Findings from the HERMOSA Intervention Study

**DOI:** 10.1289/ehp.1510514

**Published:** 2016-03-07

**Authors:** Kim G. Harley, Katherine Kogut, Daniel S. Madrigal, Maritza Cardenas, Irene A. Vera, Gonzalo Meza-Alfaro, Jianwen She, Qi Gavin, Rana Zahedi, Asa Bradman, Brenda Eskenazi, Kimberly L. Parra

**Affiliations:** 1 Center for Environmental Research and Children’s Health (CERCH), University of California, Berkeley, Berkeley, California, USA; 2 Environmental Health Laboratory, California Department of Public Heath, Richmond, California, USA; 3 Clinica de Salud del Valle de Salinas, Salinas, California, USA

## Abstract

**Background:**

Personal care products are a source of exposure to potentially endocrine-disrupting chemicals such as phthalates, parabens, triclosan, and benzophenone-3 (BP-3) for adolescent girls.

**Methods:**

We enrolled 100 Latina girls in a youth-led, community-based participatory research intervention study to determine whether using personal care products whose labels stated they did not contain these chemicals for 3 days could lower urinary concentrations. Pre- and postintervention urine samples were analyzed for phthalate metabolites, parabens, triclosan, and BP-3 using high-performance liquid chromatography/tandem mass spectrometry.

**Results:**

Urinary concentrations of mono-ethyl phthalate (MEP) decreased by 27.4% (95% CI: –39.3, –13.2) on average over the 3-day intervention; no significant changes were seen in urinary concentrations of mono-n-butyl phthalate (MnBP) and mono-isobutyl phthalate (MiBP). Methyl and propyl paraben concentrations decreased by 43.9% (95% CI: –61.3, –18.8) and 45.4% (95% CI: –63.7, –17.9), respectively. Unexpectedly, concentrations of ethyl and butyl paraben concentrations increased, although concentrations were low overall and not detected in almost half the samples. Triclosan concentrations decreased by 35.7% (95% CI: –53.3, –11.6), and BP-3 concentrations decreased by 36.0% (95% CI: –51.0, –16.4).

**Discussion:**

This study demonstrates that techniques available to consumers, such as choosing personal care products that are labeled to be free of phthalates, parabens, triclosan, and BP-3, can reduce personal exposure to possible endocrine-disrupting chemicals. Involving youth in the design and implementation of the study was key to recruitment, retention, compliance, and acceptability of the intervention.

**Citation:**

Harley KG, Kogut K, Madrigal DS, Cardenas M, Vera IA, Meza-Alfaro G, She J, Gavin Q, Zahedi R, Bradman A, Eskenazi B, Parra KL. 2016. Reducing phthalate, paraben, and phenol exposure from personal care products in adolescent girls: findings from the HERMOSA Intervention Study. Environ Health Perspect 124:1600–1607; http://dx.doi.org/10.1289/ehp.1510514

## Introduction

Cosmetics, fragrances, and other personal care products are a possible source of human exposure to potentially endocrine-disrupting chemicals, such as phthalates, parabens, and phenols (Braun et al. 2014; Meeker et al. 2013). Because women are the primary consumers of many personal care products, they are disproportionately exposed to these chemicals (CDC 2012). Adolescent girls may be at particular risk of exposure through this route. For example, one small study found that the average adult woman uses approximately 12 individual personal care products each day, whereas the average teenage girl uses 17 (Environmental Working Group 2008).

Different types of phthalates, parabens, and phenols are used in a wide variety of consumer products. The three phthalates most commonly used in personal care products are diethyl phthalate (DEP), which is found in scented products, including perfumes, deodorants, soaps, and shampoo; and di-*n*-butyl phthalate (DnBP) and di-isobutyl phthalate (DiBP), which are used in nail polish and cosmetics (Dodson et al. 2012; Guo and Kannan 2011). *In vitro* and animal studies have shown DEP, DnBP, and DiBP to have estrogenic and anti-androgenic properties (Alam et al. 2010; Harris et al. 1997; Takeuchi et al. 2005; Zhang et al. 2011), and human studies have found these phthalates to be associated with differences in behavior and allergic response in children (Braun et al. 2013). The parabens commonly used in personal care products include methyl, ethyl, butyl, and propyl paraben, which are used as preservatives and antibacterial agents in cosmetics (Soni et al. 2005). Parabens have demonstrated weak estrogenic and anti-androgenic activity in *in vitro* and rat studies (Chen et al. 2007; Routledge et al. 1998; Vo et al. 2010), although little is known about their health effects in humans. Two phenols are also commonly used in personal care products. Triclosan, an antimicrobial compound used in liquid soaps, acne cream, deodorants, shaving cream, and certain toothpastes, has been associated with alterations in thyroid hormone homeostasis in animal studies (Paul et al. 2010; Stoker et al. 2010). Benzophenone-3 (BP-3), also known as oxybenzone, is used in sunscreens, lip balm, and other sun protection products and is suspected to act as a weak estrogen based on its association with increased uterine weight in rats studies and increased proliferation of human breast cancer cells *in vitro* (Schlumpf et al. 2001).

Personal care product use is widespread, and human exposure to these chemicals is nearly ubiquitous, with mono-ester phthalate metabolites of DEP, DnBP, and DiBP detected in the urine of > 96% of Americans participating in the 2009–2010 National Health and Nutrition Examination Survey (NHANES) (Zota et al. 2014). Methyl and propyl parabens were found in > 90% of individuals, BP-3 in 97%, and triclosan in 75% (Calafat et al. 2008a, 2008b, 2010).

Awareness of endocrine disruptors in personal care products is increasing, and some companies now advertise products that are “low chemical” or “phthalate- and paraben-free.” However, to our knowledge, no studies have examined whether changing to low-chemical personal care products can lower levels of these potential endocrine disruptors in the body. Our community-based participatory research study was conducted by youth researchers in a primarily Mexican American low-income community in Northern California. The intervention aimed to determine whether adolescent girls’ urinary concentrations of metabolites of three phthalates (DEP, DnBP, and DiBP), four parabens (methyl, ethyl, butyl, and propyl paraben), and two phenols (triclosan and BP-3) decreased after switching to alternative personal care products. These nine compounds were targeted because they are widely used in personal care products; we expected to see no change in other phthalates and phenols [e.g., di(2-ethylhexyl) phthalate (DEHP), bisphenol A (BPA)] whose use is primarily in plastics and other non–personal care product consumer goods.

## Methods

The Health and Environmental Research on Makeup of Salinas Adolescents (HERMOSA) Study was a youth empowerment intervention study examining strategies to reduce personal care product chemical exposure to adolescent girls. HERMOSA means “beautiful” in Spanish, reflecting the study’s focus on Latina teens. The study was designed in collaboration with 15 local high school students (including 3 of the authors: M.C., I.A.V., G.M.-A.) participating in the CHAMACOS (Center for the Health Assessment of Mothers and Children of Salinas) Youth Council (Madrigal et al. 2014); they were hired as youth researchers and were involved in all aspects of the study, including study design, questionnaire development, identifying low-chemical replacement products, recruiting participants, collecting data, and returning results to the community.

Participants were 100 adolescent girls living in Salinas, California, a small city in an agricultural region of Northern California with a predominantly Latino population. Participants were recruited through social media, word of mouth, and personal networks of the youth researchers. Girls were eligible for the study if they were between 14 and 18 years old, spoke English or Spanish, and had lived in the United States for at least 1 year. Girls received $100 in incentive coupons for their participation plus free samples of replacement personal care products. All replacement products were selected and purchased by the study with no input from manufacturers or retailers. Data collection took place in June–July 2013. This study was approved by the Committee for the Protection of Human Subjects at UC Berkeley.

### Intervention

Girls participating in the study were provided with low-chemical personal care products and asked to refrain from using their regular products for 3 days. Each girl was provided with small (2–4 oz) polyethylene containers of shampoo, conditioner, body wash, and moisturizing lotion; a bar of hand soap (for home); a container of liquid soap (to carry in her purse); and roll-on deodorant. Additionally, each girl was allowed to choose four items from among liquid or powder foundation, mascara, eyeliner, lipstick/lip gloss/lip balm, and sunscreen. Girls were encouraged to choose the beauty products they used most often to help them comply with the intervention. Participants were asked to avoid using any personal care products or cosmetics other than those provided by the study; if a particular type of product was not provided, participants were asked to forego using that item during the intervention period. Girls who reported using Colgate Total toothpaste (the only brand used by participants that listed triclosan as an ingredient) were given alternate toothpaste; all others were allowed to use their regular toothpaste brand.

The replacement personal care products provided to participants were selected to be free of phthalates, parabens, triclosan, and BP-3. Products were not tested for the presence of these chemicals because we lacked the resources for laboratory testing and because we wanted to select products based on information available to consumers. Rather, we identified products whose ingredient lists did not include triclosan, BP-3/oxybenzone, or any parabens. Because phthalates are not listed on ingredient lists, we avoided any products containing parfum or fragrance unless they were specifically labeled as “phthalate-free.” We identified a selection of eligible soaps, shampoos, lotions, sunscreens, and cosmetics through on-line searches, consumer databases [e.g., the Skin Deep® Cosmetics Database (Environmental Working Group 2016)], and in-store research, with priority given to products that were lower cost and available at local retail stores. The final products for use in the intervention were selected by the youth researchers, who tried all the products to help identify those most likely to appeal to adolescents based on marketing, scent, and effectiveness.

### Data Collection

Participants were interviewed three times: during a home visit, and at pre- and postintervention office visits. All interviews were conducted by the youth researchers. The first visit was the home visit, during which we obtained informed assent (ages 14–17 years) or consent (age 18 years) from the participant as well as informed permission from her parent or guardian. We collected information about family income, household habits, and the family’s usual personal care and cleaning products from the parent/guardian using a brief survey. Additionally, at the home visit, we gave the participant four bins labeled “hair products,” “face products,” “body products,” and “teeth products” and asked her to place all the personal care products that she regularly used in the appropriate bin. We took photographs of each bin to ascertain brand names.

The pre- and postintervention visits occurred between 0800 and 1600 hours at the HERMOSA research office approximately 1 week after the home visit. At the preintervention visit, we measured participants’ height using a wall-mounted stadiometer and measured their weight and percent body fat using a Tanita bio-impedance scale. Participants then completed a computer-assisted, interviewer-administered questionnaire including basic demographic questions and detailed questions about personal care product use in the previous 48 hr. The participant was shown the photographs we had taken at the home visit and was asked to indicate which products she had used in the previous 48 hr and when. She was also asked if there were products she had used that were not in the pictures. She was then reminded not to use any of her usual personal care products during the intervention period. Participants provided an in-office urine sample in a sterile polypropylene cup. No wipes or external cleaning products were used before collection of the urine sample. At the conclusion of the visit, participants visited the study’s “Beauty Bar” where they selected replacement personal care products, learned about the chemicals in makeup and personal care products, and received additional instructions about avoiding all personal care products except those provided by the study for the next 3 days.

The postintervention visit was scheduled 3 days later at the same hour as the preintervention visit, to minimize the influence of diurnal variability on differences in analyte concentrations before and after the intervention. The participants provided a follow-up, in-office urine sample and completed a brief questionnaire about their knowledge, attitudes, and behaviors related to personal care product chemicals and their compliance with the intervention. They also answered open-ended questions about their experience with the intervention study.

All urine samples were aliquoted and frozen at –80°C until shipment on dry ice to the Environmental Health Laboratory of the California Department of Public Health in Richmond, California for analysis.

### Laboratory Methods

Phthalate laboratory methods were adapted from Kato et al. (2005). Ten phthalate urinary mono-ester metabolites were measured {MEP (monoethyl phthalate), MnBP (mono-*n*-butyl phthalate), MiBP (monoisobutyl phthalate), MBzP (monobenzyl phthalate), MCHP (monocyclohexyl phthalate), MEHP [mono(2-ethylhexyl) phthalate], MEHHP [mono(2-ethyl-5-hydroxyhexyl) phthalate], MECPP [mono(2-ethyl-5-carboxypentyl) phthalate], MEOHP [mono(2-ethyl-5-oxohexyl) phthalate], MCPP [mono(3-carboxypropyl) phthalate]}, although only MEP, MnBP, and MiBP (metabolites of DEP, DnBP, and DiBP, respectively) were the focus of this study. Urine samples were spiked with a mixture of stable isotope-labeled internal standards (Cambridge Isotopes) and enzymatically digested with glucuronidase at 37°C for 90 min. Five hundred microliters of digested sample solution were injected into an on-line solid-phase extraction (SPE) column and analyzed using a high-performance liquid chromatography/tandem mass spectrometer (HPLC-MS/MS) system (API 5000; AB Sciex). Target analytes were chromatographically separated on a Betasil™ phenyl column in a mobile phase consisting of acetonitrile and 0.1% acetic acid in gradient elution mode (Kato et al. 2005). Ionization of analytes was carried out with an electrospray ionization (ESI) source operating in negative mode. To enhance sensitivity, the mass spectrometer data were acquired using multi-period mode during chromatographic elution time.

The analytical method used to measure environmental phenols in urine has been previously described (Gavin et al. 2014). This method measures four parabens (methyl paraben, ethyl paraben, propyl paraben, and butyl paraben), BP-3, triclosan, and BPA. Urine samples were spiked with stable isotope-labeled internal standards and enzymatically de-conjugated overnight at 37°C. The digested samples were then processed by SPE using C18 cartridges, and the eluents were evaporated and reconstituted with mobile phase immediately before the analysis using a reverse-phase HPLC-MS/MS system (API 5500 QTRAP; AB Sciex). Ionization of the analytes was carried out by atmospheric pressure chemical ionization (APCI).

The *r* ^2^ of calibration curves for all target analytes for both methods were ≥ 0.99. The limits of detection (LOD) for phthalates were similar to or slightly higher than those used in the NHANES analysis of the general U.S. population (LODs: MEP = 0.5 μg/L, MnBP = 0.9 μg/L, MiBP = 0.4 μg/L) but were sufficiently low to detect analytes in > 97% of the study population. The LODs for phenols were generally lower than those used in NHANES (LODs: methyl and ethyl paraben = 0.5 μg/L, butyl and propyl paraben = 0.2 μg/L, triclosan = 0.2 μg/L, BP-3 = 0.5 μg/L). Randomly selected samples (*n* = 8 for the phenol panel and *n* = 11 for the phthalate panel) were analyzed in duplicate, and the relative percent differences (RPD) between duplicate results for all analytes ranged from 0 to 19.6% for samples > LOD. Quality control samples were included in every analytical run, and the recoveries were all within 30% of the respective target values. Precision for each quality control level was good, with coefficients of variation (CV) for all analytes ≤ 15%. Field quality control included collection of 20 field blanks using highly purified water treated as urine samples, including contact with all field collection materials (e.g., urine cups, aliquotting materials, vials). All target analytes in the field blanks were below the respective LODs.

Chemical analyte concentrations were reported in ng/mL of urine. Concentrations below the limit of detection were assigned the value of LOD divided by the square root of 2 (Hornung and Reed 1990).

To account for urinary dilution, urine specific gravity was measured in the field using a handheld refractometer (PAL-10S; Atago USA Inc.). Because NHANES does not report specific gravity–corrected concentrations, we also measured creatinine in the laboratory to facilitate comparison with NHANES. Creatinine was measured using applications of a colorimetric method known as the Jaffe reaction, and based on a method commercially available (BioAssay Systems QuantiChrom Creatinine Assay Kit DICT-500) but using end point reaction measurements and absorbance value collections after 45 min of incubation time at 490 nm.

### Statistical Analysis

We examined distributions of all the urinary analytes and compared them with those of all females 14–18 years of age in the 2011–2012 wave of NHANES (*n* = 108). NHANES data were downloaded from the Centers for Disease Control and Prevention website (CDC 2014). All analytes were approximately log-normally distributed, so analyte concentrations were log_10_-transformed for analysis. Geometric mean (GM) concentrations of creatinine-corrected and uncorrected analytes were compared between HERMOSA and NHANES using *t*-tests. Values < LOD were replaced with LOD divided by the square root of 2 for comparison.

We examined changes in urinary concentrations by comparing pre- and postintervention GMs and detection frequencies (DF) and used mixed-effects models to quantify average within-individual percent change in urinary concentrations before and after the intervention, controlling for time of urine collection. The primary analyses used specific gravity–corrected concentrations, calculated using the following formula: (analyte concentration × 0.024)/(sample specific gravity – 1) (Mahalingaiah et al. 2008). We also examined creatinine-corrected values and uncorrected analyte concentrations while controlling for specific gravity or creatinine. In sensitivity analyses, we ran separate mixed-effects models that *a*) excluded participants on their menses (*n* = 17), in case this contaminated the urine samples, and *b*) excluded participants who were also youth research assistants (*n* = 5), in case their involvement in the study resulted in different behaviors.

In exploratory analyses, we compared pre- and postintervention urinary concentrations of BP-3 among participants who had used sunscreen within 48 hr before the preintervention visit, and triclosan among participants who had used Colgate Total toothpaste within the 48 hr before the preintervention visit. We hypothesized that sunscreen would be the primary source of BP-3 exposure and Colgate Total would be a significant source of triclosan exposure; thus, we expected to see larger decreases when we restricted the analyses to users of these products than in the study population as a whole. Because parabens and phthalates are found in a wider range of products, we did not conduct similar exploratory analyses for these analytes.

All analyses were conducted using Stata version 13 (StataCorp, College Station, TX). Statistical significance was considered at α = 0.05.

## Results

### Demographics

The characteristics of the 100 young women enrolled in the study are shown in Table 1. All were between the ages of 14 and 18 years and self-identified as Mexican or Mexican American. More than half (57%) of participants reported speaking mostly Spanish at home, and 19% were born in Mexico. Only a third (33%) of participants reported that at least one of their parents had completed a high school education, and 38% of participants lived in households with an annual income less than the U.S. federal poverty threshold for a family of four ($24,250) (Office of the Assistant Secretary for Planning and Evaluation 2015).

**Table 1:** Characteristics of adolescent girls participating in the HERMOSA Study, Salinas, California, 2013 (*n *= 100).

Characteristic	*n* (%)
Age (years)
14	11 (11)
15	22 (22)
16	29 (29)
17	30 (30)
18	8 (8)
Country of birth
United States	81 (81)
Mexico	19 (19)
Language spoken at home
Mostly Spanish	57 (57)
Spanish and English equally	29 (29)
Mostly English	14 (14)
Highest parental education
Less than high school	57 (57)
High school graduate	33 (33)
Unknown	10 (10)
Annual household income^*a*^
≤ $24,000	38 (38)
$24,001–$36,000	29 (29)
> $36,000	25 (25)
Unknown	8 (8)
Time pre- and postintervention visit began
0800–1000 hours	33 (33)
1000–1200 hours	30 (30)
1200–1400 hours	31 (31)
1400–1600 hours	8 (8)
Frequency of makeup use
Every day	27 (27)
4–6 times per week	23 (23)
2–3 times per week	20 (20)
Once a week or less	30 (30)
Frequency of moisturizer use
Every day	65 (65)
4–6 times per week	19 (19)
2–3 times per week	8 (8)
Once a week or less	8 (8)
Frequency of fragrance use
Every day	47 (47)
4–6 times per week	18 (18)
2–3 times per week	17 (17)
Once a week or less	18 (18)
Used makeup on day of preintervention visit
Yes	67 (67)
No	33 (33)
Used sunscreen on day of preintervention visit
Yes	30 (30)
No	70 (70)
Used triclosan-containing toothpaste on day of preintervention visit
Yes	32 (32)
No	68 (68)
^***a***^Information provided by parent.	

At the time of the preintervention interview, 67% of participants were wearing makeup and 30% were wearing sunscreen. Half of the study participants (50%) reported using makeup at least 4 days per week, and 65% reported wearing fragrance at least 4 days per week. Skin moisturizer use was common, with 65% of participants reporting daily use and 84% using moisturizer at least four times per week.

### Comparison with NHANES

Exposure to the phthalates, parabens, and phenols of interest was common, with > 90% of participants having detectable concentrations of MEP, MnBP, MiBP, methyl paraben, propyl paraben, triclosan, or BP-3 in their urine at the preintervention visit (Table 2). In general, LODs tended to be higher in NHANES than in our study; in the case of triclosan, the differences were quite large (NHANES LOD = 2.3 μg/L; HERMOSA LOD = 0.2 μg/L). Creatinine-corrected urinary concentrations of all analytes except triclosan were higher among HERMOSA participants than among 14- to 18-year-old girls in NHANES, as assessed by comparing either GMs or medians. Urinary concentrations of all three phthalate metabolites were slightly higher in HERMOSA than in NHANES, but these differences were statistically significant only for MnBP (GM = 15.9 vs. 8.0 μg/g) and MiBP (GM = 8.5 vs. 6.4 μg/g). GM urinary concentrations of methyl, ethyl, butyl, and propyl paraben were significantly higher in HERMOSA participants than in NHANES (43.4 vs. 11.0; 1.6 vs. 0.9, 0.5 vs. 0.1; 12.7 vs. 1.4 μg/g, respectively), although the comparisons of ethyl and butyl parabens may be unstable due to the small proportion of girls with detectable concentrations of ethyl paraben (55% in HERMOSA, 25% in NHANES) and butyl paraben (49% in HERMOSA, 8% in NHANES). GM concentrations of BP-3 were considerably higher in HERMOSA participants (97.4 μg/g) than in NHANES adolescent girls (14.7 μg/g), although the HERMOSA samples were collected in June and July when sunscreen use is most common, whereas NHANES was conducted throughout the year. GM concentrations of triclosan were slightly lower in HERMOSA than in NHANES (5.3 vs. 8.0 μg/g) but these differences were not statistically significant. Results were similar when non–creatinine-corrected concentrations in HERMOSA and NHANES were compared, except that the difference in triclosan concentrations became statistically significant, whereas the differences in MEP, MnBP, MiBP, and ethyl paraben were no longer statistically significant (data not shown). Concentrations of BPA and the phthalates that were not the primary focus of this analysis tended to be higher in NHANES girls than in HERMOSA girls (see Table S1).

**Table 2:** Comparison of creatinine-corrected urinary analyte concentrations (μg/g) in adolescent girls participating in HERMOSA (*n *= 100) and NHANES (*n *= 108).

Analyte	HERMOSA study preintervention (2013)							NHANES girls age 14–18 years (2011–2012)							*p*-Value^*a*^
	LOD (μg/L)	DF (%)	GM (μg/g)	Percentile (μg/g)				LOD (μg/L)	DF (%)	GM (μg/g)	Percentile (μg/g)				
				25th	50th	75th	95th				25th	50th	75th	95th	
Phthalates
MEP^*b*^	0.5	100	43.9	21.33	36.4	83.7	237.9	0.4	100	29.0	15.2	31.2	62.7	267.9	0.16
MnBP^*c*^	0.9	97	15.9	11.0	15.0	22.3	46.6	1.0	96	8.0	5.1	8.8	14.6	34.0	< 0.01
MiBP^*d*^	0.4	99	8.5	5.1	7.5	13.0	32.1	0.2	100	6.4	4.3	6.7	9.9	18.7	< 0.01
Parabens
Methyl paraben	0.5	93	43.4	13.4	47.9	121.7	1,013.7	1.0	96	11.0	4.0	12.1	54.0	493.5	< 0.01
Ethyl paraben	0.5	55	1.6	< LOD	1.25	3.75	62.18	1.0	25	0.9	0.4	0.6	1.3	20.1	< 0.01
Butyl paraben	0.2	49	0.5	< LOD	< LOD	1.3	20.2	0.2	8	0.1	< LOD	< LOD	< LOD	0.6	< 0.01
Propyl paraben	0.2	90	12.7	2.83	15.35	79.7	270.71	1.0	89	1.4	0.5	1.2	6.2	89.2	< 0.01
Phenols
Triclosan	0.2	93	5.3	1.0	3.7	17.5	579.2	2.3	82	8.0	2.0	5.0	26.7	261.6	0.17
BP-3	0.5	97	97.4	26.6	117.2	434.3	2,938.2	0.4	100	14.7	3.8	9.2	30.1	375.7	< 0.01
Abbreviations: DF, detection frequency; GM, geometric mean; LOD, limit of detection. ^***a***^*t*-Test comparison of HERMOSA and NHANES geometric means, replacing values < LOD with LOD divided by the square root of 2. ^***b***^Metabolite of diethyl phthalate.^***c***^Metabolite of di-*n*-butyl phthalate. ^***d***^Metabolite of di-isobutyl phthalate.															

### Impact of Intervention on Chemical Exposure

Geometric mean urinary concentrations of phthalate metabolites, paraben, triclosan, and BP-3 all decreased over the course of the 3-day intervention (Table 3). From the mixed-effects models controlling for time of day, we observed an average decrease of 27.4% [95% confidence interval (CI): –39.3, –13.2] in MEP metabolite concentrations (preintervention GM = 78.2 μg/L vs. postintervention GM = 56.4 μg/L; *p* < 0.001) and a nonsignificant 11.3% (95% CI: –22.2, 1.1) decrease in MnBP concentrations (preintervention GM = 28.3 μg/L vs. postintervention GM = 25.1 μg/L; *p* = 0.07). There were no statistically significant changes in MiBP urinary concentrations. Metabolite levels decreased in 68%, 58%, and 55% of girls for MEP, MnBP, and MiBP, respectively (Table 3). Figure S1 illustrates the individual changes pre- and postintervention for each girl, showing that metabolite levels increased for some girls. The proportion of girls with phthalate concentrations < LOD did not change significantly pre- and postintervention, with all three phthalates detected in almost all girls’ urine at both time points.

**Table 3:** Change in specific gravity–corrected concentrations (ng/mL) of urinary analytes before and after the HERMOSA intervention.

Analyte	Preintervention		Postintervention		Percent change (95% CI)^*a*^	Girls with decrease (%)
	DF (%)	GM (SE)	DF (%)	GM (SE)		
Phthalates
MEP	100	78.2 (1.1)	99	56.4 (1.1)	–27.4 (–39.3, –13.2)	68
MnBP	97	28.3 (1.1)	98	25.1 (1.1)	–11.3 (–22.2, 1.1)	58
MiBP	99	15.2 (1.1)	99	15.2 (2.3)	–0.5 (–12.6, 13.3)	55
Parabens
Methyl paraben	93	77.4 (1.2)	87	43.2 (1.2)	–43.9 (–61.3, –18.8)	61
Ethyl paraben	55	2.9 (1.2)	63	4.2 (1.2)	47.3 (–0.7, 118.4)	45
Butyl paraben	49	0.8 (1.2)	62	1.7 (1.2)	101.7 (35.5, 203.2)	39
Propyl paraben	90	22.6 (1.3)	87	12.3 (1.2)	–45.4 (–63.7, –17.9)	63
Phenols
Triclosan	93	9.5 (1.3)	90	6.1 (1.2)	–35.7 (–53.3, –11.6)	65
BP-3	97	173.8 (1.2)	97	113.4 (1.2)	–36.0 (–51.0, –16.4)	65
Abbreviations: DF, detection frequency; GM, geometric mean; SE, standard error. ^***a***^From mixed-effects model adjusting for time of urine collection (using 24-hr clock hours and minutes).						

Methyl and propyl paraben, the most commonly detected parabens, decreased by 43.9% (95% CI: –61.3, –18.8) and 45.4% (95% CI: –63.7, –17.9) on average, respectively (Table 3). The GM of methyl paraben decreased from 77.4 μg/L to 43.2 μg/L. The proportion of girls with detectable concentrations of methyl paraben decreased nonsignificantly from 93% to 87%, and decreases in concentrations were observed in 61% of girls. The GM of propyl paraben decreased from 22.6 μg/L to 12.3 μg/L, with decreases observed in 63% of girls; the proportion of girls with detectable concentrations of propyl parabens also decreased between pre- and postintervention (90% vs 87%), but not significantly. Unexpectedly, ethyl and butyl paraben concentrations both increased over the course of the intervention period, with butyl paraben increasing by 101.7% (95% CI: 35.5, 203.2) and ethyl paraben increasing by a nonsignificant 47.3% (95% CI: –0.7, 118.4), on average. However, the absolute changes in concentrations were small for both butyl paraben (preintervention GM = 0.8 μg/L vs. postintervention GM = 1.7 μg/L) and ethyl paraben (preintervention GM = 2.9 μg/L vs. postintervention GM = 4.2 μg/L), and these findings should be interpreted with caution because these analytes were detected only in about half of participants. Although detection frequencies increased postintervention, the differences were not statistically significant. Individual patterns in changes in paraben concentrations are shown in Figure S1.

Triclosan concentrations decreased by 35.7% (95% CI: –53.3, –11.6) over the 3-day intervention (preintervention GM = 9.5 μg/L vs. postintervention GM = 6.1 μg/L) (Table 3). The decrease was larger when we restricted our analysis to the 32 girls who reported using triclosan-containing toothpaste at the preintervention visit; among these girls, triclosan concentrations decreased by 70% (preintervention GM = 36.4 μg/L vs. postintervention GM = 11.0 μg/L) (data not shown). Overall, triclosan concentrations decreased in 65% of girls. BP-3 concentrations decreased by 36.0% (95% CI: –51.0, –16.4) from a preintervention GM of 173.8 μg/L to a postintervention GM of 113.4 μg/L. When we restricted the analysis to the 30 girls who reported using sunscreen at the preintervention visit, BP-3 concentrations decreased by 52% (preintervention GM = 434.4 μg/L vs. postintervention GM = 203.0 μg/L). BP-3 concentrations decreased in 65% of girls, overall. No significant differences were seen in the proportion of participants with detectable levels of triclosan or BP-3 pre- and postintervention. Changes in analyte concentrations for each individual are shown in Figure S1.

Results comparing pre- and postintervention analyte concentrations were similar using creatinine-corrected rather than specific gravity–corrected values, and the decrease in MnBP became statistically significant (see Table S2). Results did not change substantially when creatinine or specific gravity was included as a covariate in the mixed-effects model rather than using creatinine- or specific gravity–corrected values or when girls on their menses or girls employed as youth research assistants were excluded (data not shown).

Urinary concentrations of other measured analytes that are not primarily found in personal care products, such as the phthalate metabolites MBzP, MCHP, MEHP, MEHHP, MECPP, MEOHP, and MCPP and BPA, did not change during the course of the intervention (see Table S3).

### Participant Compliance

Compliance with the intervention varied for different product types (Table 4). At the postintervention visit, > 90% of participants reported that they used only the low-chemical replacement hair, makeup, and body products they were given. However, 35% of participants stated that, at least once during the 3 day intervention, they had washed with soaps other than the replacement soaps provided by the study. The main reasons for noncompliance were forgetting (32%), being away from home (12%), and using their regular product in cases when they did not choose a replacement among the additional four products allowed (21%).

**Table 4:** Participants’ compliance and behaviors related to personal care product use in HERMOSA Study.

Compliance/behavior	Percent
Used only HERMOSA replacement products during intervention period
Hair products	91
Makeup	91
Face products	90
Scented products	94
Body products	94
Soap products	65
Among participants with any noncompliance during intervention (*n *= 57): Why did you use non-HERMOSA products?
Forgot	32
Was away from home	12
Didn’t get a replacement product^*a*^	21
Replacement product didn’t work	2
Didn’t like look	0
Didn’t like smell	0
Knowledge and attitudes
Learned something new about chemicals in cosmetics	66
Has checked beauty products for phthalates, parabens, triclosan, oxybenzone	23
Will buy products without phthalates, parabens, triclosan, oxybenzone	71
^***a***^Either this particular replacement product was not offered or the participant did not choose it among her four discretionary replacement products.	

### Changes in Knowledge, Attitudes, and Behaviors

Most girls stated that they had learned something new about chemicals in cosmetics because of the study (66%) and that they would buy products without phthalates, parabens, triclosan, or BP-3/oxybenzone (71%) (Table 4). At the postintervention visit, 23% of girls reported that they had checked the ingredients of their regular products for these chemicals. Participants seemed to enjoy being in the study. Several girls stated that they appreciated learning about chemical exposures in personal care products and being taught methods for reducing their exposure (Appendix 1).

## Discussion

The adolescent girls in this study experienced an average within-girl decline of 27–45% in urinary concentrations of certain phthalates, certain parabens, triclosan, and oxybenzone after 3 days of abstaining from conventional personal care products and using replacement products with labels indicating they did not contain these chemicals.

Of the classes of chemicals studied, we found that phthalate concentrations decreased the least, with a 27% average decrease in MEP, an 11% nonsignificant average decrease in MnBP, and no change in MiBP. The relatively modest change in these compounds over the intervention period may be attributable to the presence of these phthalates in other scented products in the home that were not replaced during the intervention, including air fresheners, fabric softeners, and cleaning products (Buckley et al. 2012; Cacho et al. 2015; Viñas et al. 2015) as well as ingestion of phthalates in food (Colacino et al. 2010) and medications (Hernández-Díaz et al. 2013; Kelley et al. 2012). It is estimated that the majority of an adult woman’s cumulative exposure to DEP is through personal care product use, but that her cumulative exposure to DnBP and DiBP may also reflect significant uptake from food and house dust (Guo and Kannan 2013). This is consistent with our finding of a significant decrease in MEP but not the other two metabolites. Additionally, MnBP is slower to clear from the body after exposure than MEP (Janjua et al. 2008a), so it is possible that 3 days was not long enough to detect statistically significant changes in MnBP concentrations. Finally, there are indications that DEP and DnBP use in personal care products is decreasing, presumably due to consumer advocacy (Campaign for Safe Cosmetics 2011). Several cosmetics companies have pledged to remove phthalates from their products (Kessler 2015), and urinary concentrations of MEP and MnBP have decreased substantially in the general U.S. population since 2004 (Zota et al. 2014), suggesting that the contribution of personal care products to Americans’ overall phthalate exposure may be declining.

The compounds that declined the most during the intervention were methyl and propyl paraben, which decreased by 44–45% on average over the course of our study. Parabens can be found in a wide variety of personal care products including cosmetics, lotions, soaps, and shampoos (Guo and Kannan 2013). Although several parabens are also used as preservatives in baked goods and processed food (Soni et al. 2005), levels are lower than in personal care products, and diet is not considered to be a major source of exposure (Liao et al. 2013). Dermal absorption from personal care products is likely the primary route of exposure (Guo and Kannan 2013; Liao et al. 2013), which is consistent with the reductions in urinary paraben concentrations seen in this study.

Contrary to expectations, we observed increases in urinary concentrations of ethyl and butyl parabens during the intervention, although the absolute levels of these compounds remained low. The ingredient lists of all the replacement beauty products indicated that they were paraben-free, though it is possible that some of these products contained ethyl and butyl paraben either as an unintentional contaminant or as an unlabeled substitute for methyl and/or propyl paraben. A limitation of our study is that we were not able to test the replacement products to ensure that they did not contain the chemicals of concern, so we were unable to identify the sources of the increased ethyl and butyl paraben exposure.

We also observed average decreases of 36% in triclosan and BP-3 concentrations. Triclosan exposure is thought to occur by ingestion among users of triclosan-containing toothpaste and by dermal absorption among users of antibacterial soaps, skin cleansers, and other triclosan-containing personal care products (CDC 2013b). We found that the decrease in triclosan concentrations was largest among girls who had been using toothpaste containing triclosan but changed brands during the study, suggesting that toothpaste may be a significant source of triclosan exposure. BP-3 is an ultraviolet-protection agent found mainly in sunscreens and cosmetics offering sun protection (CDC 2013a), which is consistent with our finding of the largest decrease in BP-3 levels among sunscreen users.

The phthalates, parabens, and phenols examined in this study are nonpersistent compounds with short half-lives that are cleared from the body within 24–48 hr following exposure (Gonzalez et al. 2006; Janjua et al. 2008a, 2008b; Sandborgh-Englund et al. 2006), which allowed us to examine average differences in urinary concentrations after just 3 days. We measured the analytes in spot urine samples, which reflect very recent exposure, likely over the past 12 to 24 hr. Collecting 24-hr urine samples from the participants in this study would have yielded a more comprehensive picture of exposure over that entire day, but would have imposed considerably more burden on the participants. By collecting the spot samples at the same time of day before and after the intervention, we minimized the influence of within-day variability while assessing the impact of the intervention on recent exposure.

Although urinary concentrations of MEP, methyl and propyl paraben, triclosan, and BP-3 decreased over the course of the intervention, they were not eliminated. Even after changing to low-chemical products, the majority of girls (> 90%) continued to have detectable concentrations of these compounds in their urine. One possibility is that not all of the preintervention exposure was cleared from the body during the intervention period; particularly for BP-3, there is evidence that low levels of the chemical may remain in the body for up to 5 days after dermal exposure (Gonzalez et al. 2006). Thus, it is possible that the 3-day intervention period was too short and that we might have observed larger decreases in urinary metabolite concentrations if the girls had used the replacement personal care products for a longer period of time. We selected the 3-day intervention period because we assumed it was long enough to detect changes in urinary metabolite levels but short enough to maximize participant compliance. It is also likely that other sources of exposure beyond personal care products exist, and/or that the replacement products were not completely free of these compounds. Although on average most analytes decreased, many participants experienced increases in some analyte levels over the course of the intervention. Upon receiving individual results, some of these girls were surprised that their levels had risen despite their best efforts to comply. Their experience highlights the difficulty of reducing exposure to these ubiquitous compounds.

HERMOSA Study participants, who were examined in 2013, had urinary concentrations of the chemicals of interest that were similar to or slightly higher than adolescent girls participating in NHANES in 2011 and 2012. It is unlikely that the differences in LODs between HERMOSA and NHANES accounted for much of the observed differences in urinary concentration between the two populations, because median as well as geometric mean levels tended to be higher in HERMOSA. The NHANES and HERMOSA samples were analyzed by different laboratories, which may affect comparability. However, the California Department of Public Health laboratory that analyzed the HERMOSA samples has participated in and passed the proficiency test program administrated by the CDC every year (two rounds per year) since 2012 to ensure that their results are comparable with those of NHANES. Additionally, although concentrations of parabens, MiBP, MnBP, and BP3 were higher in HERMOSA participants, concentrations of other phthalates and phenols not primarily found in personal care products were higher in NHANES girls, suggesting that the differences were attributable to exposure patterns rather than systematic analytical differences between laboratories. We compared the HERMOSA urinary concentrations with those of all adolescent girls in NHANES, rather than Latina adolescents, because of NHANES sample size limitations. The HERMOSA Study participants were all Latina and mostly low-income and their personal care product use patterns may differ from than the general U.S. population, which may affect the generalizability of our findings. The finding of higher analyte concentrations in our population are consistent with studies in NHANES showing that levels of phthalates and parabens are higher among African Americans and Mexican Americans than whites (CDC 2012).

Overall, compliance was good in this study (> 90% for all products except hand soap), although most girls found it difficult to completely comply with the protocol, particularly regarding use of hand soap. Presumably decreases in exposure would have been larger with perfect compliance. Most girls stated that they would like to use products without potentially harmful chemicals. However, identifying these products often requires a considerable amount of effort and expense on the part of the consumer. In our experience, we had little trouble purchasing low-chemical hand soaps and shampoos even in mainstream retailers, but found that low-chemical makeup was hard to identify, not sold many places, and often expensive. Although many stores have dedicated displays for low-chemical personal care products, these still represent only a small portion of the personal care product market and, in our experience, can be particularly difficult to find in low-income communities. Future studies should look at the long-term effects of education about endocrine disruptors in personal care products on girls’ product choices, behavior changes, and exposure levels.

Finally, a major strength of this study was that it was rooted in the tenets of community-based participatory research, including bidirectional learning between the researchers and community youth. Involving the youth research assistants in the study design and implementation allowed us to create an intervention that was culturally appropriate, scientifically valid, and relevant and interesting to the adolescent participants. The 100% retention, good compliance, and detailed data collection were likely attributable to the connection that the youth research assistants were able to forge with the study participants.

In summary, this is the first study that we know of to show that techniques available to consumers, such as choosing personal care products that are labeled as free of phthalates, parabens, triclosan, and oxybenzone, can significantly reduce personal exposure to these potentially endocrine-disrupting chemicals. Our study did not test for the presence of these chemicals, but simply used techniques available to the average consumer: reading labels and investigating product safety through web-based databases. Our findings suggest that consumers may be able to reduce exposures by seeking out commercially available products with lower levels of these chemicals.

Appendix 1 **Comments from HERMOSA Participants**

What did you enjoy most about participating in the HERMOSA study?

I enjoyed meeting the members of HERMOSA study and getting introduced to other products of makeup that can be healthier to your skin.
Realizing that the powder HERMOSA study provided me was not as heavy as the makeup brand I usually used and looked more natural.
What I enjoyed the most was challenging myself to not use my products and just the products I was given.
Trying more natural products and learning about the harsh chemicals found in the usual products I use.
I enjoyed learning [about] low-chemical products. This was a good experience and a cool challenge most people should participate in to become informed on how their everyday products are affecting them.
I enjoy getting FREE makeup.
I enjoyed feeling naturally beautiful.

How has the study been beneficial to you?

The study has taught to make smarter choices by choosing healthier and low chemical products.
I learned that you can look good and still keep track of what your products contain.
It has been beneficial because I learned that there are harmful things in the products we use every day.
The study [was] beneficial to me because now I know what products I can buy for better health.
This study has been beneficial to me in that it made me aware that products we have at home serve the same function as other products that are found in the store.
The study has been beneficial to me because now I’m more aware of the chemicals my makeup has.

It actually made me want to buy these [low chemical] products on my own because I really liked them.

## Supplemental Material

(1.5 MB) PDF
