# Supplementary material for: Reducing Phthalate, Paraben, and Phenol Exposure from Personal Care Products in Adolescent Girls: Findings from the HERMOSA Intervention Study
Source: Environ Health Perspect. 2016 Mar 7;124(10):1600–7. doi: 10.1289/ehp.1510514 (PMC5047791; doi:10.1289/ehp.1510514)

Environ Health Perspect
DOI: 10.1289/ehp.1510514

Note to readers with disabilities: *EHP* strives to ensure that all journal content is accessible to all readers. However, some figures and Supplemental Material published in *EHP* articles may not conform to 508 standards due to the complexity of the information being presented. If you need assistance accessing journal content, please contact ehp508@niehs.nih.gov. Our staff will work with you to assess and meet your accessibility needs within 3 working days.

Supplemental Material

Reducing Phthalate, Paraben, and Phenol Exposure from Personal Care Products in Adolescent Girls: Findings from the HERMOSA Intervention Study

Kim G. Harley, Katherine Kogut, Daniel S. Madrigal, Maritza Cardenas, Irene A. Vera, Gonzalo Meza-Alfaro, Jianwen She, Qi Gavin, Rana Zahedi, Asa Bradman, Brenda Eskenazi, and Kimberly L. Parra

Table of Contents

Table S1. Creatinine-corrected urinary concentrations (µg/g) of other phthalates and phenols not primarily found in personal care products, comparing adolescent girls participating in HERMOSA (N=100) and NHANES (N=108)
Table S2. Pre- and post-intervention creatinine-corrected urinary concentrations (ng/g)
Table S3. Pre- and post-intervention specific-gravity-corrected urinary concentrations (ng/mL) of other phthalates and phenols not primarily found in personal care products
Figure S1. Individual pre- and post-intervention specific-gravity-corrected urinary concentrations (ng/mL) of A) mono-ethyl phthalate (MEP), B) MEP, enlarged to focus on values <600 ng/ml, C) mono-n-butyl phthalate (MnBP), D) mono-isobutyl phthalate (MiBP), E) MiBP, enlarged to focus on values <200 ng/mL, F) methyl paraben, G) ethyl paraben, H) butyl paraben, I) propyl paraben, J) triclosan and K) benzophenone-3 (BP-3) analytes for each participant in the study.

Table S1. Creatinine-corrected urinary concentrations (µg/g) of other phthalates and phenols not primarily found in personal care products, comparing adolescent girls participating in HERMOSA (N=100) and NHANES (N=108)

	HERMOSA Study Pre-Intervention 2013							NHANES 2011-2012 (Girls 14-18)							
	LOD	DF	GM	Percentile (µg/g)				LOD	DF	GM	Percentile (µg/g)				
Analyte	(µg/L)	(%)	(µg/g)	25%	50%	75%	95%	(µg/L)	(%)	(µg/g)	25%	50%	75%	95%	p-value[a]
Phthalates:															
MBzP	0.2	100	4.9	2.8	4.6	7.8	22.2	0.3	99	5.3	3.2	5.5	10.1	20.6	0.50
MEHP	0.2	88	1.4	.72	1.6	2.4	6.1	0.5	78	1.4	.61	1.2	2.8	10.2	0.96
MEHHP	0.2	98	4.7	3.0	4.3	7.9	15.9	0.2	100	8.4	4.6	7.0	10.6	53.9	<0.01
MECPP	0.2	100	8.7	5.7	8.1	12.3	33.6	0.2	100	13.6	7.2	10.5	18.1	65.1	<0.01
MEOHP	0.1	99	3.7	2.4	3.6	5.5	12.5	0.2	100	5.3	3.0	4.3	7.1	31.6	0.02
MCPP	0.1	97	1.4	0.9	1.4	1.9	4.3	0.2	98	2.9	1.1	1.8	4.8	45.7	<0.01
Phenols:															
BPA	0.2	81	0.8	.45	.9	1.5	4.3	0.4	91	1.6	.74	1.2	2.3	8.7	<0.01

[a]Comparison of HERMOSA and NHANES geometric means
Abbreviations: LOD = Limit of detection, DF = Detection Frequency, GM = Geometric Mean

Environ Health Perspect
DOI: 10.1289/ehp.1510514

Table S2. Pre- and post-intervention creatinine-corrected urinary concentrations (ng/g)

	Pre-intervention	Post-intervention	Mixed Effect Model[a]	
Analyte	GM (SE)	GM (SE)	% change (95% CI)	p-value
Phthalates:				
MEP	43.9 (1.1)	31.4 (1.1)	-28.3 (-39.6, -14.9)	<0.001
MnBP	15.9 (1.1)	14.0 (1.1)	-11.9 (-20.5, -2.4)	0.02
MiBP	8.5 (1.1)	8.5 (2.3)	-0.2 (-9.9, 10.4)	0.96
Parabens:				
Methyl paraben	43.4 (1.2)	24.1 (1.2)	-44.5 (-61.1, -20.8)	<0.01
Ethyl paraben	1.6 (1.2)	2.4 (1.2)	43.5 (-2.6, 111.7)	0.07
Butyl paraben	0.5 (1.2)	0.9 (1.2)	100.1 (32.8, 201.6)	<0.01
Propyl paraben	12.7 (1.2)	6.9 (1.2)	-45.6 (63.3, -19.3)	<0.01
Phenols:				
Triclosan	5.3 (1.3)	3.4 (1.2)	-36.3 (-53.2, -13.4)	<0.01
BP-3	97.4 (1.2)	63.2 (1.2)	-35.1 (-49.7, -16.1)	<0.01

[a]Adjusted for time of urine collection(using 24-hour clock hours and minutes).

DOI: 10.1289/ehp.1510514

Table S3. Pre- and post-intervention specific-gravity-corrected urinary concentrations (ng/mL) of other phthalates and phenols not primarily found in personal care products

	Pre-intervention	Post-intervention	Mixed Effect Model[a]	
Analyte	GM (SE)	GM (SE)	% change (95% CI)	p-value
Phthalates:				
MBzP	8.7 (1.1)	8.8 (1.1)	1.1 (-6.9, 9.7)	0.80
MEHP	2.4 (1.1)	2.8 (1.1)	15.9 (-3.4, 39.0)	0.11
MEHHP	8.3 (1.1)	8.4 (1.1)	1.2 (-13.8, 18.8)	0.88
MECPP	15.6 (1.1)	16.5 (1.1)	5.4 (-9.3, 22.6)	0.49
MEOHP	6.7 (1.1)	6.8 (1.1)	1.7 (-13.0, 18.8)	0.84
MCPP	2.6 (1.1)	2.4 (1.1)	-7.3 (-23.5, 12.2)	0.44
Phenols:				
BPA	1.4 (1.1)	1.6 (1.1)	10.6 (-12.8, 40.3)	0.40

[a]Adjusted for time of urine collection (using 24-hour clock hours and minutes).

Environ Health Perspect
DOI: 10.1289/ehp.1510514

Figure S1. Individual pre- and post-intervention specific-gravity-corrected urinary concentrations (ng/mL) of A) mono-ethyl phthalate (MEP), B) MEP, enlarged to focus on values <600 ng/ml, C) mono-n-butyl phthalate (MnBP), D) mono-isobutyl phthalate (MiBP), E) MiBP, enlarged to focus on values <200 ng/mL, F) methyl paraben, G) ethyl paraben, H) butyl paraben, I) propyl paraben, J) triclosan and K) benzophenone-3 (BP-3) analytes for each participant in the study.

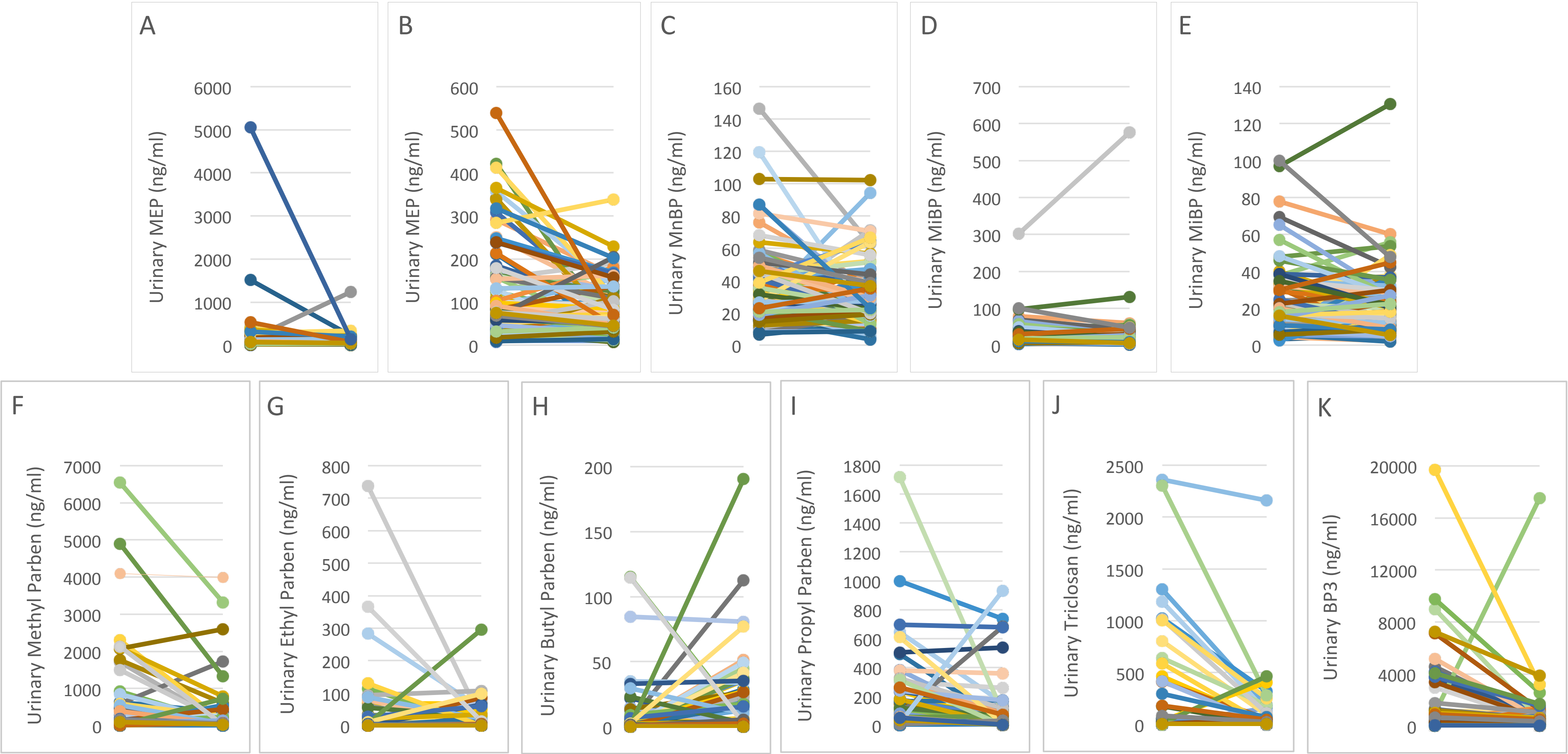

Supplement: (1.5 MB) PDF [file ehp.1510514.s001.acco.pdf]
